# Differing Pattern of Ambulatory Blood Pressure in Very Elderly Men Expresses Dynamics in Atherosclerotic Load in the Senescence

**DOI:** 10.1155/2012/417291

**Published:** 2011-12-18

**Authors:** Arkadiusz Siennicki-Lantz, Sölve Elmståhl

**Affiliations:** Division of Geriatric Medicine, Department of Health Sciences, Skane University Hospital in Malmö, Lund University, SE-205 02 Malmö, Sweden

## Abstract

To assess an impact of vascular risk factors on ambulatory blood pressure measurement (ABPM) in the elderly, we followed up a population-based cohort of men from 68 until 82 years, when 104 survivors underwent ABPM. *Results*. At age 68, hypertension and high clinic blood pressure (CBP) did not predict ABPM level. Smoking and low ankle-brachial index (ABI) predicted higher ABPM variability and pulse pressure (PP), but not absolute ABPM values. At age 82, hypertension, high or increasing CBP, strongly positively correlated with all variables of ABPM. Carotid stenosis, low or declining ABI during followup, correlated with higher nocturnal ABPM and PP. *Concluding*. Hypertension and vascular risk factors in a cohort of 68-year-old men do not result in higher ABPM at age 82, possibly due to inflection point in their pressure development. Higher ABPM reflects instead an increasing CBP and aggravating atherosclerosis during the preceding decade in that part of the cohort with previously favorable risk factor status.

## 1. Introduction

Blood pressure levels in the very elderly are more scattered than in younger elderly or middle-aged persons. After initial blood pressure increase, which occurred up to the seventh decade in both sexes, a blood pressure decline has been observed [1, 2]. High initial blood pressure level was typical for elderly subjects with subsequent BP decline [3]. Furthermore, levels of blood pressure in the very elderly have paradoxical inverse relationship to morbidity and mortality. The most described covariates and consequences of blood pressure decline have been shorter survival [4–6], cognitive decline [7, 8], and dementia [9–12]. Heart studies showed that demented patients had lower blood pressure and thinner left ventricle posterior wall [13]. Cognitive impairment was also common in subjects with heart failure combined with hypotension [14, 15].

Studies describing ambulatory blood pressure (ABPM) are mainly focused on younger elderly or middle-aged persons, mainly with essential hypertension, and seldom comprising population-based samples [16–20]. Frequency of sustained, white-coat, and reverse hypertension in the very elderly is also unknown. In most study centers, a profile of ABPM in younger elderly or middle-aged persons was used as a predictor of vascular events later in their life. In the very elderly, level of ABPM should be regarded not only as a predictor of target organ damage, but also as a mirror of general vascular status.

The aim of our study was to assess a profile of ABPM in a cohort of octogenarian men who were longitudinally followed since random inclusion from a population of city of Malmö, Sweden. Contrary to previous studies, we assessed an impact of vascular and life-style risk factors observed at age 68, and a time progress of atherosclerotic disease, on the ABPM profile when subjects reached the age of 82 years.

## 2. Methods

### 2.1. Study Sample

**Figure 1:**
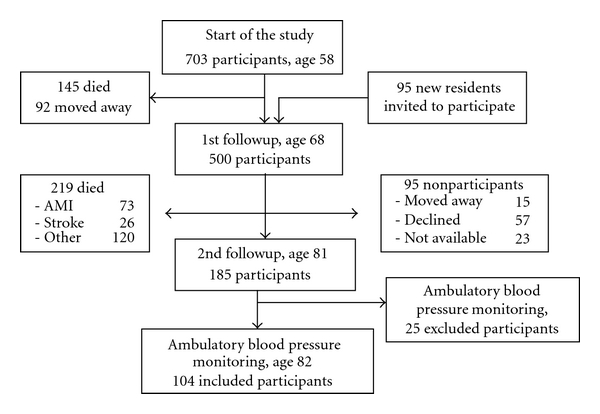
Follow-up of the cohort, “Men born in 1914”.

A prospective population sample study, “Men born in 1914”, has been in progress since 1968. It includes all men born in the even months of 1914 in the city of Malmö, Sweden. A total of 809 men were invited to participate in the study, and 703 men took part in the first health examination. When they were 68 years old, 465 men in the cohort and additional 95 new residents were invited to attend a new examination. Five hundred of them agreed to participate (Figure 1). The most recent followup of the cohort started when the subjects reached 81-82 years of age, and 281 men were found to be still alive. Of these, 185 agreed to take part (66%) in a new investigation, including both physical and psychological examinations. Blood pressure data and psychological data were available from 171 of them at the ages of 68 and 81. In the following year, 129 subjects underwent ambulatory blood pressure monitoring (ABPM). 25 subjects were excluded according to ABPM quality criteria. 104 subjects were included into the final statistical analysis. 

### 2.2. Health Examination

Study subjects and their spouses answered to a questionnaire focusing on life-style factors, prescribed medicines, and previous diseases. All underwent medical examination including Hachinski ischemic score. To evaluate the role of established vascular risk factors, we measured levels of blood glucose, cholesterol, and triglycerides during fasting conditions and body mass index (BMI) at age 68. The participants were also classified as nonsmokers, former smokers, and smokers. Tobacco consumption of the smokers was measured as g/day. Alcohol consumption was self-reported and calculated in g 100% ethanol per week. At the recent followup at age 81, the medical examination was repeated, and 185 men answered a questionnaire focusing on lifestyle and health markers. Possible dementia was classified according to the DSM-IV criteria, and one subject was diagnosed as being demented.

Two established markers of vascular disease were examined: carotid stenosis, determined using carotid ultrasound at age 81, and low peripheral circulation in the lower extremities, estimated using the ankle-brachial pressure index (ABI) at ages 68 and 81. 

### 2.3. Blood Pressure Measurement

The clinic blood pressure (CBP) was measured sphygmomanometrically in the upper right arm, in the supine position after 15 min of rest at age 68 and at age 81, using a calibrated mercury manometer and rubber cuffs (12 × 35 cm for normal, and 15 cm for obese subjects). Hypertension was defined as systolic and diastolic brachial BP ≥160 mmHg or ≥90 mmHg, respectively, or medication for hypertension. These hypertension criteria have been used previously and were valid until the World Health Organization drew up new ones in 1999 [21]. All the subjects had been monitored and treated during their lifetime according to these hypertension criteria, and they were thus used for the statistical analysis in this study.

### 2.4. Ambulatory Blood Pressure Monitoring at Age 82

Ambulatory blood pressure monitoring was performed using Micro AM Recorder, Model KI5600 (Kontron Instruments). Readings at 20-minute intervals during a day (from 06.20 AM to 09.40 PM) and at 60 min intervals at night (from 10.00 PM to 06.00 AM) were performed. Monitoring was performed in patient's private environment without specific advices regarding physical activity. The ambulatory BP-measurement was performed with auscultatory method, but in case of measurement failure the examination was immediately repeated using an oscillometric method. The accuracy of KI5600 was confirmed by a simultaneous measurement with a standard mercury sphygmomanometer and accepted if they were within 10 mmHg of standard method. The exclusion of patients was made according to the quality criteria: deficit in measurement time intervals at least 6 h accumulated during a daytime or more than 3 h accumulated at nighttime, or more than 3 h consecutively during a daytime or at least 2 h consecutively during a nighttime. For the individual data, the relative nocturnal BP fall was calculated using a formula: (daytime BP-nighttime BP) × 100/daytime BP, and expressed in %. Preawakening SBP was defined as a mean of measurements at 04.00, 05.00, and 06.00 AM. Postawakening SBP was a mean of measurements: 06.20, 06.40, 07.00, 07.20, 07.40, and 08.00. Morning SBP surge is defined as a difference between Postawakening SBP and Mean SBP nighttime.

### 2.5. Peripheral Arterial Circulation at Age 68 and 81

Ankle blood pressure was estimated, both at ages 68 and 81 years, by placing a cuff at the ankle level and using Doppler signal on tibial posterior artery or dorsal foot artery to detect peripheral blood flow in the supine position. Reference pressure in the arm was calculated using strain gauge recording system. Arithmetic average of duplicate recordings was used. For each leg, an ankle-brachial pressure index (ABI) was calculated by dividing the ankle systolic pressure with the highest upper arm systolic pressure value.

### 2.6. Carotid Duplex Ultrasonography

The examination of carotid arteries was made at age 81, using computed sonography system (Acuson XP 10, Acuson, Mountain View, Calif, USA) with a 7 MHz B-mode real-time linear scanner, including a 5 MHz-pulsed and color-coded Doppler. The color-coded Doppler was used to localize areas with high-flow velocities in the internal carotid artery, and the maximum-flow velocity (m/s) was measured with the pulsed Doppler.

### 2.7. Statistics

Summary values are expressed as mean ± standard deviation. Correlation analyses were performed using Spearman correlation test. Differences in vascular risk factors/markers were calculated with Mann-Whitney rank sum test. All data analysis has been performed using SPSS (SPSS Inc., Chicago, IU, USA) statistical package. A two-tailed *P *value of less than 0.05 was considered statistically significant. Local ethical committee at Lund University accepted the study, and informed consent was obtained from all participants.

## 3. Results

Values of ABPM, that is, daytime and nighttime SBP, DBP, systolic and diastolic variability (mean SD-SBP and SD-DBP), nocturnal SBP fall, morning SBP surge, preawakening SBP, and postawakening SBP are presented in Table 1. Levels of clinic blood pressure, P-cholesterol, triglycerides, and B-glucose as well as markers of vascular disease, that is, carotid stenosis, ankle-brachial index, and its time-change during follow-up, are also presented.

### 3.1. ABPM Levels Compared to Previously Published Results on Younger Elderly Persons

Compared to a sample of 70-year-old population of Uppsala, Sweden [22], subjects of this study had lower mean daytime SBP with 9 mmHg, mean DBP, and PP with 5 mmHg but the same variability/standard deviation. At nighttime, data of this cohort and Uppsala cohort were similar concerning SBP, DBP, and PP.

Compared to a sample (age 73 ± 6) from a population of Madrid, Spain [23] at daytime, mean values of SBP, DBP, PP, and SD were very similar with only 1-2 mmHg differences. The same was observed if compared to nighttime values, except for nighttime PP which was lower with 5 mmHg in this study.

Compared to 15-year younger Japanese population-based sample of Ohasama study (mean age 66.7 y) [24], subjects of this study had similar daytime SBP and DBP but higher nighttime SBP with mean 5 mmHg.

Compared to Italian Pamela population study (mean age 69.0 y ± 2.3), subjects of this study had similar daytime values, higher nighttime SBP with 6 mmHg but not DBP [25, 26].

Compared to the oldest sample of population-based study from Dublin, Ireland (age 50–79 y) [27], subjects of this study had lower daytime SBP with 2 mmHg, daytime DBP with 4 mmHg, higher nighttime SBP with 7 mmHg, and equal mean nighttime DBP. Daytime values of the Irish study were similar to this study in the sample at age 40–49 y.

Compared to Uruguayan population sample of men untreated for hypertension at age >70 y [28], subjects of this study had lower mean daytime SBP with 3 mmHg, DBP with 5 mmHg, and mean nighttime SBP with 3 mmHg but similar nighttime DBP. Compared to younger elderly (50–59 and 60–69 y), subjects of this study had similar daytime and nighttime SBP but lower daytime DBP with 7 mmHg and nighttime DBP with 3 mmHg.

In a population study from Denmark [29], several small subgroups in different age intervals were studied. Compared to the subgroup at age 70–79 y, subjects of this study had lower daytime SBP with mean 7 mmHg, daytime DBP with 3 mmHg, higher nighttime SBP with 3 mmHg, and lower DBP with 2 mmHg. Compared to the subgroup at age 60–69 y, subjects of this study had lower daytime SBP with mean 12 mmHg, daytime DBP with 10 mmHg, the same nighttime SBP, and lower DBP with 3 mmHg. Compared to the subgroup at age 50–59 y, subjects of this study had lower daytime SBP with mean 3 mmHg, daytime DBP with 5 mmHg, higher nighttime SBP with 6 mmHg, and lower DBP with 2 mmHg. Small samples of the Danish study were presented by high standard deviation of each BP value.

### 3.2. Does Hypertension at Age 68 or 81 Predict ABPM Levels?

Hypertension, diagnosed or treated during the first followup at age 68, has been tested as possible predictor of ABPM 14 years later (Table 2, right columns). The values of ABPM did not differ between subjects who were hyper- and normotensive at age 68. When hypertension was defined with the same criteria at age 81 (Table 2, left columns), the values of ABPM examined the same year differed between the groups and presented, in hypertensive subjects, higher daytime SBP and PP, and higher nocturnal SBP and PP, and higher pre- and postawakening SBP, but did not differ concerning relative morning surge or diurnal BP variability.

### 3.3. Does Time Course of Clinic BP between Age 68 and 81 Predict ABPM Levels?

**Figure 2:**
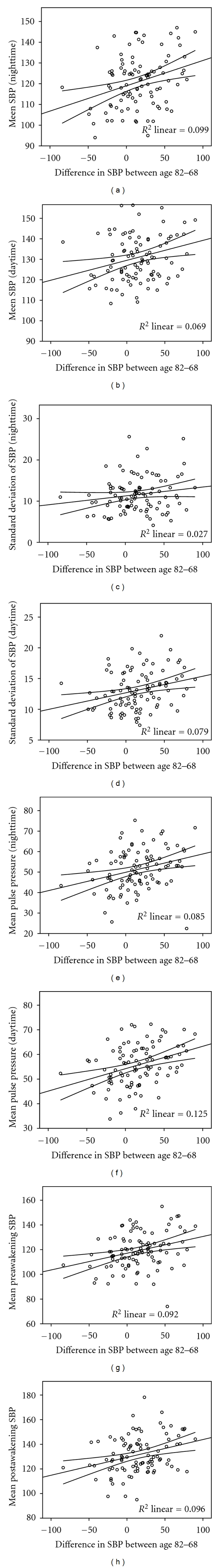
Correlation between an arithmetic difference in SBP, measured at ages 81 and 68 and ambulatory blood pressure measures collected at age 82 (daytime, nighttime, pre- and postawakening SBP, SBP variability, that is, daytime and nighttime standard deviation of SBP measurements and daytime and nighttime pulse pressure). Positive difference in SBP means an increasing SBP during the followup.

We have previously shown that blood pressure dynamics differed in these study subjects during the followup. Those, who presented higher clinic BP levels at age 68, were prone to have declining SBP until age 81 [30]. In this study, time course of SBP correlated positively with mean SBP and daytime, nighttime, and with pre- and postawakening SBP levels (Figure 2). Nighttime SBP was strongest correlated with increasing clinic SBP. High ambulatory pulse pressure reflected also increasing clinic SBP over time. Highest daytime SBP variability was observed in subjects with increasing office SBP.

### 3.4. Do Vascular Risk Factors and Markers of Atherosclerosis at Age 68 Predict ABPM Values at Age 82?

To estimate the impact of vascular risk factors at age 68 on future ABPM levels, we calculated if there was a correlation between office BP, levels of P-cholesterol, triglycerides, glucose at age 68, and ABPM levels 14 years later (Table 3), without recording any significant values. However, BMI levels at age 68 correlated negatively with daytime DBP and its variability, that is, SD-DBP. In addition, ABI levels at age 68 correlated negatively with future SBP variability and with pulse pressure at daytime, presenting the lowest ABI levels in subjects with highest daytime SBP variability and pulse pressure. ABPM values have been splitted according to their smoking profile at age 68 (Table 4). Those subjects who were still current smokers at age 68 had higher systolic and diastolic pressure variability (SD-SBP, SD-DBP) both daytime and nighttime. The absolute values of SBP or DBP did not differ between these groups, neither daytime nor nighttime.

### 3.5. Does ABPM Reflect Clinic BP (CBP) and Markers of Atherosclerosis at Age 81?

At age 82, the CBP correlated positively with daytime: SBP, SD-SBP, DBP, and PP, and with nighttime: SBP, DBP, and PP as well as with pre- and postawakening SBP (Table 5). Clinic DBP was expressed better by daytime SBP and DBP levels, than clinic SBP. No correlation was observed with nocturnal SBP fall or morning SBP surge. Carotid stenosis correlated positively with nocturnal and preawakening SBP and daytime PP, but not with daytime SBP or DBP values. Ankle-brachial index was lowest in subjects with higher nocturnal: SBP, PP, SBP variability, and preawakening SBP. Daytime BP values did not correlate with ABI. The time course of ABI between age 68 and 82 showed that the largest ABI decline was reflected by higher daytime and nighttime systolic variability, that is, SD-SBP, and by higher PP, as well as by higher pre- and postawakening SBP levels, but not by daytime or nighttime SBP/DBP levels at age 82.

## 4. Discussion

This study provides a longitudinal observation data on a population-based sample of elderly men between ages of 68 and 82 years. The baseline data of ABPM performed in the study subjects at age 82 should be discussed in the light of other population-based samples. The majority of previously published studies on ABPM included either preselected hypertensive elderly patients or examined younger elderly populations. Compared to latter studies performed in cohorts aged 70–79 y, octogenarians from our study had generally lower daytime levels of SBP/DBP and in some cases even lower nighttime SBP/DBP levels. Our ABPM levels were similar to those registered in men in their 50–60-ties. In the Danish study [29], a similar profile of increasing ABPM values in the younger samples until age of 70 y was observed, but a decreasing ABPM in a subgroup at age 80+. This age-related threshold of ABPM values could be supported by our observation that the higher level of office-BP or suffering from hypertension at age 68 did not predict higher ABPM neither daytime nor nighttime at age 82. Instead, longitudinal change in Clinic BP during 13 years correlated with ABPM values. By analyzing ABPM values in hypertensive and normotensive subjects at age 82, we could conclude that low values of ABPM in the whole cohort were partly due to low ABPM values in those men who were hypertensive at age 68, and at the same time developed decline of Clinic BP until their 80-ties. On the other side, higher values of ABPM were observed not in those subjects who were highly hypertensive at 68 but those who developed hypertension in the last decade and had largest increase in vascular burden during that time.

These observations could be confirmed by the data showing that established laboratory risk factors at age 68 did not predict future levels of ambulatory blood pressure. However, lower ankle-brachial index and particularly current smoking at age 68 predicted larger BP variability both daytime and nighttime. Yet, when measured at age 81, Clinic BP and being diagnosed as hypertensive at age 81 could be strongly reflected by higher values of ABPM and especially diurnal pulse pressure (PP). ABPM could also adequately express the grade of atherosclerotic process at age 82 by higher nighttime and preawakening BP-levels, higher nighttime BP variability, and PP values, in those men who had higher grade of carotid stenosis, lower ABI, and extended ABI-decline during the 14-year followup.

Possible explanation of the lower ABPM values in the very elderly, compared to the younger population samples, could be a selective mortality of those subjects from our cohort, who died before age 68, that is, before the first followup, due to early hypertension, metabolic syndrome, intensive smoking, and advanced atherosclerosis [31]. Another explanation could be the fact that survivors, who were included in this sample, had been less exposed to vascular risk factors than those who declined to take part in the last followup or died prior to it. However, in the whole examined sample, SBP decreased with mean 7 mmHg and DBP with 9 mmHg, which points to the fact that not only selective mortality is an explanatory factor, but also a part of the cohort expresses a BP decline during the last 14 observation years, which results in lower ABPM levels compared to the younger population.

Cigarette smoking, as the strongest risk factor at age 68, did not predict absolute values of BP in octogenarians, but increasing SBP and DBP variability (SD) by ca 20%, both daytime and nighttime. Similarly, lower ABI level at age 68 predicted higher daytime SBP variability and PP, and not the absolute ABPM values.

At age 81, subjects defined as hypertensive expressed higher nighttime and daytime SBP, post- and preawakening SBP, and above all, higher PP. The values of clinic BP at age 81 correlated with values of ABPM, mainly clinic DBP, high values which were reflected by higher daytime and nighttime SBP, DBP, SD-SBP, and pre- and postawakening SBP as well as daytime PP. Clinic SBP at age 81 was reflected only by nighttime SBP and PP. It suggests that in very elderly men clinic DBP seems to express overall 24-h BP profile in a more adequate way that clinic SBP, and that diurnal PP should be used as an important complement to both clinic and diurnal BP measurements.

Nocturnal values of ABPM could be used as a risk factor or marker of vascular burden in the octogenarian men. Nighttime and preawakening SBP and daytime PP correlated best with a grade of carotid stenosis. Similar result were observed concerning ABI, where high nighttime SBP, SD-SBP, PP, and high preawakening SBP were observed in subjects with a diminished peripheral leg circulation. Daytime values did not express that risk. The largest progress in peripheral arterial disease, expressed as a decreasing ABI over 14 years, was observed not in these subjects who had high absolute ABPM values, but in those who expressed high PP and high SBP-variability both night- and daytime, and had larger pre- and postawakening SBP.

## 5. Conclusion

In conclusion, in a population sample cohort of 82-year-old men, high daytime and nighttime ABPM measurements reflected increasing office-BP and aggravating atherosclerosis only in the last decade. Subjects with early developed hypertension, peripheral atherosclerosis and active smokers already in their 60 ties reached an inflection point in their blood pressure development and did not express increasing ABPM values in their eighties any longer.

## Figures and Tables

**Table 1:** The background data from the 1st and the 2nd followup of the cohort “Men born 1914”.

	Age 68 years	Age 81-82 years
Smoking (*n *active/*n* former or never smoked)	28 versus 76	
BMI	24.5 (17.4)	
B-glucose	4.9 (.52)	
P-Cholesterol	6.0 (.94)	
P-triglycerides	1.4 (.66)	
Ankle-brachial index right	1.11 (.11)	.99 (.20)
Ankle-brachial index left	1.07 (.13)	.96 (.21)
Difference ABI-R age 82–68		−.13 (.17)
Difference ABI-L age 82–68		−.11 (.17)
*Clinic BP (mmHg) *		
Systolic	151.1 (19.9)	144.1 (15.4)
Diastolic	92.2 (10.3)	83.1 (6.2)
*Ambulatory BP (mmHg)*		
Daytime, average BP		
Systolic		131.1 (12.0)
Diastolic		75.5 (10.4)
Pulse pressure		55.6 (8.2)
Nighttime, average BP		
Systolic		120.9 (12.7)
Diastolic		67.5 (10.9)
Pulse pressure		51.4 (9.7)
Average standard deviation of:		
Daytime systolic		13.1 (3.0)
Daytime diastolic		10.0 (2.9)
Nighttime systolic		11.7 (4.2)
Nighttime diastolic		9.5 (3.4)
Nocturnal SBP fall (%)		7.7 (6.1)
Morning SBP surge (mmHg)		26.3 (16.2)
Preawakening, average SBP		119.2 (14.5)
Postawakening, average SBP		131.3 (15.2)

**Table 2:** Ambulatory blood pressure values measured in elderly men at age 82, who were diagnosed hypertensive versus normotensive during examinations at age 81 and 68 years.

	Hypertension at 81			Hypertension at 68		
	Yes (*n* = 60)	No (*N* = 44)		Yes (*n* = 59)	No (*N* = 45)	
*ABPM at age 82*	Median min–max	Median min–max	*P*	Median min–max	Median min–max	*P*
*Daytime (mmHg)*						
SBP	132.5 114.9–159.9	126.0 108.4–144.7	.006**	129.1 108.4–159.4	130.0 109.1–159.9	.651
SD-SBP	13.2 7.4–21.9	12.4 7.9–19.7	.180	12.9 7.4–21.9	13.0 7.9–19.7	.580
DBP	72.4 57.8–104.7	75.8 55.3–96.5	.979	71.9 57.8–104.8	76.5 55.3–99.9	.433
SD-DBP	9.5 4.6–18.7	9.5 4.7–15.8	.942	9.3 4.6–18.7	9.3 4.7–16.3	.909
PP	58.7 47.0–72.3	52.4 33.8–72.3	.000**	55.6 33.8–72.0	54.7 36.2–72.3	.759
*Nighttime (mmHg)*						
SBP	124.4 95.0–145.0	114.4 94.0–147.0	.011*	122.0 94.0–144.7	121.4 101.6–147.0	.552
SD-SBP	11.8 5.2–25.1	10.7 4.1–25.6	.120	11.4 4.1–25.6	10.9 5.3–25.2	.826
DBP	66.3 50.6–102.5	65.4 50.2–93.9	.382	64.7 50.2–102.5	66.3 51.0–93.9	.268
SD-DBP	9.2 2.5–21.3	9.1 3.2–21.7	.679	9.2 3.2–21.7	9.3 2.5–20.2	.224
PP	53.3 22.4–71.7	49.1 25.7–75.3	.023*	48.8 22.4–75.3	53.7 34.9–74.7	.184
Nocturnal SBP fall (%)	7.8 −8.3–17.7	7.4 −7.1–19.8	.430	7.0 −7.1–16.8	8.7 −8.3–16.8	.854
Morning SBP surge (mmHg)	27.7 −.67–56.2	24.1 −2.6–123.0	.139	26.3 −2.6–51	24.5 6–123	.972
Preawakening SBP	120.2 92.0–155.0	113.5 74.0–147.0	.011*	119.3 92.3–155	116.5 74–147	.592
Postawakening SBP	132.5 107.8–78.2	124.9 95.0–155.0	.010*	127.6 97.7–166	130.0 95–178.2	.438

**Table 3:** Correlation coefficients calculated for ambulatory blood pressure at age 82 and vascular risk factors (BMI, laboratory levels and clinic blood pressure/BP) as well as for markers of vascular disease at age 68 (ABI: ankle-brachial index).

	BMI	Laboratory levels,			Clinic BP		Ankle-brachial index	
		Glucose	Triglycerides	Cholesterol	SBP	DBP	Right	Left
*Daytime*								
SBP	−.096	.015	.022	−.066	.047	−.092	.036	−.051
SD-SBP	−.082	−.026	.117	−.022	.087	−.052	−.265**	−.246*
DBP	−.210*	−.186	.005	−.035	.023	−.184	.091	.125
SD-DBP	−.237*	−.092	.008	−.038	.034	−.161	−.035	.003
PP	.052	.151	−.033	−.091	−.019	.086	−.084	−.212*
*Nighttime*								
SBP	−.019	−.050	.099	−.014	.057	−.035	.071	−.011
SD-SBP	.006	.058	.032	.031	−.092	−.146	−.100	.016
DBP	−.093	−.127	.092	−.026	.013	−.103	.082	.088
SD-DBP	.021	−.027	.104	.078	.081	−.012	−.078	.089
PP	.101	.064	.026	−.114	−.111	−.097	.113	−.049
Nocturnal SBP fall	−.104	.053	−.151	−.114	−.030	−.072	−.81	−.075
Morning SBP surge	−.014	−.005	−.105	−.111	−.139	−.023	−.025	−.139
Preawakening SBP	−.014	−.067	.018	−.088	.066	.010	.042	−.045
Postawakening SBP	−.043	−.110	−.026	−.119	−.062	−.034	.120	−.049

**Table 4:** Difference in ambulatory blood pressure at age 82 between subjects defined as current and never/former smokers *at age 68. *

	Smoking status at age 68				
	Current smokers		Never and former		
	(*N* = 28)		(*N* = 76)		
*ABPM at age 82*	Median	min–max	Median	min–max	*P*
* Daytime (mmHg)*					
SBP	131.2	108.4–159.9	128.9	109.1–159.4	.43
SD-SBP	14.5	8.6–21.9	12.6	7.4–19.7	.012*
DBP	77.4	59.7–99.9	72.7	55.3–104.7	.18
SD-DBP	11.6	7.8–17.7	9.2	4.6–18.7	.003**
PP	55.2	33.8–72.0	55.4	36.2–72.3	.75
* Nighttime (mmHg)*					
SBP	123.5	102.1–147.0	119.2	94.0–145.0	.28
SD-SBP	13.1	4.1–18.5	10.6	5.3–25.6	.023*
DBP	66.6	53.7–93.9	64.9	50.2–102.5	.45
SD-DBP	10.2	6.1–21.7	8.8	2.5–21.3	.019*
PP	53.4	37.1–70.6	52.1	22.4–75.3	.36
Nocturnal SBP fall (%)	6.6	−8.3–17.9	8.6	−7.1–19.8	.52
Morning SBP surge (mmHg)	21.6	−2.6–45.7	26.2	−.25–123.0	.15
Preawakening SBP	119.7	92.3–155.0	118.0	74.0–147.3	.33
Postawakening SBP	125.2	97.7–166.0	129.3	95.0–178.2	.35

**Table 5:** Correlation coefficients calculated for ambulatory blood pressure at age 82 and clinic blood pressure as well as for markers of vascular disease at age 81 (ABI: ankle-brachial index, ABI progression, and carotid stenosis at ultrasound examination).

	Carotid ultrasound	Clinic BP age 81		Ankle-brachial index		Ankle-brachial index	
						difference age 81–68	
	Mean stenosis	SBP	DBP	Right	Left	Left	Right
* Daytime*							
SBP	.157	.169	.341**	−.092	−.191	−.124	−.190
SD-SBP	.128	.000	.226*	−.147	−.183	−.264**	−.063
DBP	.003	−.005	.218*	.002	−.068	.051	−.095
SD-DBP	.004	−.038	.176	−.010	−.135	−.077	−.041
PP	.234*	.228**	.215*	−.192	−.174	−.234*	−.210*
* Nighttime*							
SBP	.194*	.198*	.264**	−.100	−.230*	−.141	−.181
SD-SBP	.070	.132	.036	−.220*	−.319**	−.219**	−.180
DBP	.040	.101	.267**	−.009	−.126	−.016	−.063
SD-DBP	−.083	−.006	−.051	−.018	−.182	−.051	−.032
PP	.174	.287**	.029	−.209*	−.268**	−.194*	−.315**
Nocturnal SBP fall	−.022	.000	.131	.026	.133	.058	.008
Morning SBP surge	−.024	.093	.069	−.125	.000	−.073	−.167
Preawakening SBP	.194*	.148	.259**	−.117	−.255**	−.204*	−.157
Postawakening SBP	.100	.162	.243*	−.141	−.212*	−.144	−.266**
